# Endobronchial one-way valves for treatment of persistent air leaks: a systematic review

**DOI:** 10.1186/s12931-017-0666-y

**Published:** 2017-11-06

**Authors:** Mei Ding, Ya-dong Gao, Xian-Tao Zeng, Yi Guo, Jiong Yang

**Affiliations:** 1 Department of Respiratory Medicine, Zhongnan Hospital of Wuhan University, Donghu Road 169, Wuhan, 430071 People’s Republic of China; 2 Center for Evidence-based and Translational Medicine, Zhongnan Hospital of Wuhan University, Donghu Road 169, Wuhan, 430071 People’s Republic of China

**Keywords:** Persistent air leak, Bronchopleural fistulas, Alveolar-pleural fistulas, Endobronchial valve, Bronchoscopic intervention

## Abstract

Persistent air leak (PAL) is associated with significant morbidity and mortality, prolonged hospitalization and increased health-care costs. It can arise from a number of conditions, including pneumothorax, necrotizing infection, trauma, malignancies, procedural interventions and complications after thoracic surgery. Numerous therapeutic options, including noninvasive and invasive techniques, are available to treat PALs. Recently, endobronchial one-way valves have been used to treat PAL. We conducted a systematic review based on studies retrieved from PubMed, EMbase and Cochrane library. We also did a hand-search in the bibliographies of relevant articles for additional studies. 34 case reports and 10 case series comprising 208 patients were included in our review. Only 4 patients were children, most of the patients were males. The most common underlying disease was COPD, emphysema and cancer. The most remarkable cause was pneumothorax. The upper lobes were the most frequent locations of air leaks. Complete resolution was gained within less than 24 h in majority of patients. Complications were migration or expectoration of valves, moderate oxygen desaturation and infection of related lung. No death related to endobronchial one-way valves implantation has been found. The use of endobronchial one-way valve adds to the armamentarium for non-invasive treatments of challenging PAL, especially those with difficulties of anesthesia, poor condition and high morbidity. Nevertheless, prospective randomized control trials with large sample should be needed to further evaluate the effects and safety of endobronchial one-way valve implantation in the treatment of PAL.

## Background

Pulmonary air leak is a common clinical problem which can be resulted from both bronchopleural fistulas (BPFs) and alveolar-pleural fistulas (APFs). BPFs are abnormal communications between the bronchial tree and the pleural space, while APFs are pathologic communications between the alveoli and pleural space [1]. Persistent air leak (PAL) is defined as those lasting more than 5 to 7 days postoperatively, without forced expiration or expulsion maneuvers [2, 3]. The presence of PAL is associated with significant morbidity and mortality, prolonged hospitalization and increased health-care costs [4].

Air leak, both BPF and APF, can arise from a number of conditions, including, but not limited to, pneumothorax, necrotizing infection, trauma, malignancies, procedural interventions (biopsy, CPR, radiofrequency ablation of lung tumors, etc) and complications after thoracic surgery [5–7]. Primary spontaneous pneumothorax rarely results in PAL; it is substantially more frequent (20%) in the setting of underlying COPD [8]. Incidence of iatrogenic pneumothorax is reported at 1.36% in hospitalized patients due to invasive procedures or positive pressure ventilation [9]. More commonly, lung resections carry significant risks of PALs, with an incidence ranging from 8% after a sublobar resection to 45% after lung volume reduction surgery (LVRS). The incidence of postoperative air leak ranges from 28% to 60% immediately after surgery, 26% to 48% on postoperative day 1, 22% to 24% on day 2, and 8% on day 4 [10–12]. The National Emphysema Treatment Trial suggests that postoperative air leak occur in 90% of patients undergoing bilateral LVRS procedures [13].

Numerous therapeutic options, including noninvasive and invasive techniques, are available to treat PALs. Noninvasive approaches rely on prolonged chest tube drainage either on water seal or Heimlich valve system or coupled with tailored ventilator strategies to establish acceptable ventilation while reducing the flow through the alveolar- or bronchopleural fistula. Invasive therapies include pleurodesis either surgical or at bedside through the indwelling chest tube, by instillation of talc slurry or doxycycline, mechanical pleurodesis by pleural abrasion, application of fibrin sealant, bronchial stump stapling, muscle flap construction, omental flap coverage, or pericardial fat pad flap to the bronchial stump, and surgical lobectomy [5, 14].

Recently, endobronchial one-way valves, initially designed for bronchoscopic lung volume reduction (BLVR) in emphysema, have been used to treat PAL. The objective of this article was to summarize current clinical evidence of the management of PAL with endobronchial one-way valves to define the role of this bronchoscopic intervention.

## Methods

### Literature search strategy

A search was conducted on PubMed, EMbase and Cochrane library for original studies published from 2005 to April 2017 on endobronchial one-way valves placement for treating PAL, using the keywords as “valve” AND “air leak” OR “bronchopleural fistula” OR “alveolar-pleural fistula”. We also did a hand-search in the bibliographies of relevant articles for additional studies.

### Selection criteria

Studies reporting data on endobronchial one-way valves in the treatment of PAL were included in this review. Abstracts, animal studies and studies published in languages other than English and German were excluded from this review.

### Definitions

Persistent air leak was defined as the presence of an air leak lasting more than 5 days with postsurgical or medical etiology. In studies where a different definition was used, we adopted the definition specified by the authors of the selected papers.

## Results

### Description of included studies

**Fig. 1:**
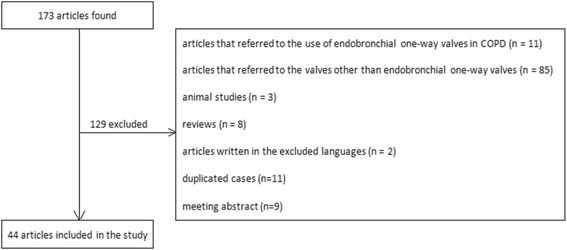
Study flowchart. One hundred and seventy-three full-length articles were identified by our literature search and 129 were excluded

One hundred and seventy- three full-length articles were identified by our literature search. After being appraised, 129 were excluded due to the following reasons: articles that referred to the use of endobronchial one-way valves in COPD (*n* = 11), articles that referred to the valves other than endobronchial one-way valves (*n* = 85), animal studies (*n* = 3), reviews (n = 8), articles written in the excluded languages (*n* = 2) and duplicated cases (n = 11), meeting abstract (*n* = 9). The search strategy was showed in Fig. 1.

### Case reports of PAL treated with endobronchial one-way valves

Characteristics of included case reports are shown in Table 1, data for 52 patients from 34 case reports are available [15–48]. Age of patients ranged from 18 to 93 years with median age at 57 years. Most of the patients were males (28/51, 54.9%). The most common underlying diseases in patients were lung infection (11/52, 21.2%), cancer (14/52, 26.9%) and lung related diseases (26/52, 50%) which included bronchiectasis, COPD, cystic fibrosis, bulla and lymphangioleiomyomatosis.

**Table 1:** Characteristics of patients and endobronchial one-way valve deployment in case reports included

	n/N	Percentage
Demographics		
Age (years), median (range)	57 (18–93)	
Male sex	28/51^a^	54.9%
Female sex	23/51^a^	45.1%
Underlying diseases		
Lung infection	11/52	21.2%
Infections of other sites	1/52	2%
Lung related diseases	26/52	50%
Chest trauma	1/52	2%
Cancer	14/52	26.9%
Systematic diseases	3/52	5.8%
Causes of air leak		
Pneumothorax	23/52	44.2%
Tension pneumothorax	6/52	11.5%
Iatrogenic pneumothorax	6/52	11.5%
Broncho-cutaneous fistula	1/52	2%
Bronchopleural fistula	6/52	11.5%
Alveolar-pleural and trans-diaphragmatic fistula	1/52	2%
Empyema	9/52	17.3%
Postoperative air leak	12/52	23.1%
Duration of air leak before endobronchial one-way valve deployment (days),median (range)	15, (3–2520)	
Location of air leak		
Right upper	17/52	32.7%
Right middle	6/52	11.5%
Right lower	6/52	11.5%
Left upper	15/52	28.8%
Left lower	8/52	15.4%
Right main bronchus	1/52	2%
Left main bronchus	1/52	2%
Lingual	5/52	9.6%
Bronchus intermedius	1/52	2%
Type of endobronchial one-way valve used		
Emphasys®	32/122	26.2%
Spiration® IBV valves	59/122	48.4%
Zephyr®	31/122	25.4%
Number of endobronchial one-way valves (per patient),median (range)	2, (1–8)	
Duration of air leak after endobronchial one-way valve deployment		
< 1 day	31/50^b^	62%
1 day≦ ≦2 days	6/50^b^	12%
> 2 days	13/50^b^	26%
Outcomes		
Removal of endobronchial one-way valve	20/50^c^	40%
Migration of endobronchial one-way valve	1/50^c^	2%
Expectoration of endobronchial one-way valve	1/50^c^	2%
Recurrence of air leak	3/50^c^	6%
Death not related to deployment of endobronchial one-way valve	3/50^c^	6%

^a^sex of patient in one case report not given

^b^duration of initial air leak not given in 2 case reports

^c^1 patient lost to follow-up and 1 patient without plan to valve removal

Clinical features of air leak: the most remarkable cause was pneumothorax (tension pneumothorax comprised) (35/52, 67.3%). Postoperative air leak and empyema were following significant causes of air leak with percentage at 23.1% and 17.3% respectively. The median duration of air leak before EBV deployment was 15 days. Air leak was most frequently located in upper lobes as 17/52, 32.7% for right upper lobe and 15/52, 28.8% for left upper lobe. It was least located in main bronchi (2/52, 4%) and bronchus intermedius (1/52, 2%). The rest lobes shared an average location of air leak.

Information of endobronchial one-way valves: the median number of valves deployed in patients was 2. Three commercial types of valves were used: Emphasys® (32/122, 26.2%), Spiration® IBV valves (59/122, 48.4%) and Zephyr® valves (31/122, 25.4%).

Outcome of endobronchial one-way valves deployment: optimal decrease of air leak was gained posterior valve deployment within less than 24 h in majority of patients (31/50, 62%). Valves were removed after cessation of air leak in 20/50 patients (40%). One case of valve migration and another case of valve expectoration were described. Recurrence of air leak was reported in 3 patients. Three patients died after valve deployment but not related to the procedure. None of complication like infection was noted.

### Case series of PAL treated with endobronchial one-way valves

Characteristics of included case series are shown in Table 2 and Table 3, data for 156 patients from 10 case series were identified in which half were prospective studies [14, 49–57]. Four children patients were reported while the rest were adults. Most of the patients were males (96/143, 67.1%). The most common underlying disease was COPD, emphysema and cancer. The largest amount of endobronchial one-way valves were deployed in the upper lobes with 28.4% (75/264) of overall at right upper lobe and 26.5% (70/264) at left upper lobe. Complete resolution was obviously noted in all case series with rate ranging from 47.5% to 100%. Ninety (57.7%) patients received removal of endobronchial one-way valves and the mean duration of endobronchial one-way valve use was 23–138 days. Recurrence was observed in 14/156 (9.0%) patients. Travaline et al. reported 6 patients with complications related to endobronchial one-way valves in their case series in 2009: valve expectoration, moderate oxygen desaturation, initial malposition of the valve redeployment required, pneumonia, methicillin-resistant *Staphylococcus aureus* colonization and one unspecified. Hance et al. reported 2 deaths after endobronchial one-way valve deployment but not related to the procedure. There was no other complication reported in the rest 148/156 (94.9%) patients.

**Table 2:** Characteristics of patients and endobronchial one-way valve deployment in case series included

First author, year [Article no.]	Publication type	No. of patients, sex(M/F)	Age (years)	Underlying disease (n/N)	Previous interventions (n/N)	Duration of air leak before valve placement	Location of endobronchial one-way valves (n/N)	Type of endobronchial one-way valves; No. of valves used
Travaline, 2009 [49]	Retrospective study	40 (25/15)	Mean ± SD: 60 ± 14	Cancer (12/40), COPD(12/40), pneumonia (3/40), rheumatoid arthritis(2/40), tuberculosis(1/40), trauma(1/40), aspergilloma(1/40), bronchiectasis (1/40), cor pulmonale(1/40), lung transplantation (1/40), multiple comorbidities (1/40)	chest tube (39/40), Eloesser flap (1/40), Blood patch (3/40), wedge resection (1/40), pleurodesis (1/40)	Median: 20 days; Interquartile range [IQR]: 15 to 45 days	RUL (11/40), RML (3/40), RLL (3/40), LUL (11/40), LLL(5/40), RUL and RLL (1/40), RLL and RML (1/40), RUL and LUL (1/40);	Zephyr®; Mean ± SD: 2.9 ± 1.9
Gillespie, 2011 [50]	Retrospective study	7 (4/3)	Median:58; Range: 17 to 60	Emphysema (5/7), neoplasia (3/7), pulmonary embolism (2/7), radiation fibrosis (1/7), fungal infection (1/7), empyema (1/7)	surgical interventions (6/7); pleurodesis or pleurectomy (4/7)	Median: 4 weeks; Range: 2 weeks to 5 months	LUL (4/31); LLL (6/31); RUL (7/31); LUL and LLL (14/31)	IBV Valve; Median: 3.5
Firlinger, 2013 [51]	Prospective study	13	Not specified	Empyema (4/13), pulmonary metastasis (1/13), pleural mesothelioma (3/13), pneumothorax (2/13), lung cancer (2/13), bronchiectasis (1/13)	Chest tube (4/13), lobectomy (3/13), decortication (2/13), radical pleurectomy (3/13), pleurodesis (1/13)	Median: 17 days; Range: 8–31 days	LUL (2/19), LLL (5/19), RUL (10/19), RLL (2/19)	IBV Valve (13/19) and Zephyr® (6/19), Mean ± SD: 1.4 ± 0.7
Reed, 2015 [52]	retrospective study	21 (11/10)	Range: 16 months to 70 years	postoperative (8/21), pneumothorax (11/21), cavitary lung infection (3/21), post-pneumonectomy bronchopleural fistula (2/21), COPD(3/21), Pneumonia(4/21), Rheumatoid lung disease(1/21), ARDS after cardiac surgery (121/), Lung cancer(1/21), Refractory AML(21/), Hypoxic cardiac arrest(121/), Refractory multiple myeloma(1/21)	Chest tube(18/21), Pleurodesis(1/21), Decortication(1/21), intercostal muscle flap coverage (2/21)	Median: 8 days; Mean, 26 days; Range: 1 to 192 days	RUL(20/88), RML(8/88), RLL(8/88), LUL(21/88), Lingula(6/88), LLL(12/88), 2 patients not specified	IBV Valve; Median:3; Range: 1 to 12
Dooms, 2014 [53]	prospective study	10 (9/1)	Median:67; Range: 46 to 75	Lung cancer (10/10)	Chest tube (10/10), chemical pleurodesis (not specified)	Median: 7 days; Range: 7 to 13 days	RLL (17/42), RUL (10/42), LLL (15/42)	IBV Valve; Median: 4; Range:1 to 9
Cordovilla, 2015 [54]	prospective study	8 (7/1)	Mean: 68.5 Range:28 to 85	severe pulmonary emphysema(7/8), respiratory failure (5/8), thrombopenia (1/8), ischemic heart disease (1/8) cancer(1/8)	Chest tube (8/8)	Median: 15.5 days	RLL (2/9), Lingula (1/9), RUL (3/9), LUL (1/9), LLL (2/9)	IBV valves(8/9) and Zephyr® (1/9); Median: 2; Range: 1 to 4
Hance, 2015 [55]	retrospective study	14 (10/4)	Mean: 60	non-small cell lung cancer (4/14), diffuse interstitial lung disease (1/14), cytomegalovirus pneumonitis (1/14), COPD (4/14), histoplasmosis (1/14), pneumonia (1/14), pulmonary nodule (1/14), idiopathic pulmonary fibrosis (1/14), aspergillosis (1/14), cystic fibrosis (1/14), metastatic mesothelioma (1/14), spontaneous pneumothoraxes (3/14)	Chest tube (14/14)	Mean: 21.6 days; Median: 18 days;	Not specified	IBV Valve (14); Median: 2; Range: 1–8
Podgaetz, 2015 [56]	retrospective study	19 (12/7)	Median: 60; Mean:60.4;Range: 38–90	Osteosarcoma (1/19), COPD (9/19), angiosarcoma (1/19), cryptogenic organizing pneumonia (1/19), pancreatic cancer (1/19), interstitial lung disease (2/19)	Chest tube (19), Chemical pleurodesis (2), Blood patch (1)	Median: 9 days; Mean: 12.8 days; Range: 2–8 days	LUL + lingula (1), RUL (5), LUL (7), RLL (3), RML (1), Lingula (2)	IBV Valve (72); Median: 4; Range: 2–6
Bakhos, 2016 [57]	prospective study	11 (9/2)	Mean ± SD: 65 ± 15; Range: 33–83	Lung cancer (5/11), mesothelioma (1/11), COPD (6/11), coccidiomycosis (1/11), interstitial lung disease (2/11)	Chest tube (11)	Mean ± SD:16 ± 12 days	RLL (3), RUL (3), LUL (3), RML (1), RUL and RML (1)	IBV Valve; Median: 2; Range: 1–4
Podgaetz, 2016 [14]	prospective study	13 (9/4)	Median: 60; Mean:61.9;Range: 38–90	Osteosarcoma (1/13), COPD (7/13), Iatrogenic (4/13), Postoperative (2/13), Angiosarcoma (1/13), pancreatic cancer (1/13), cryptogenic organizing pneumonia(1/13)	Chest tube (13)	Median: 9 days; Mean: 14.9 days; Range: 2–88 days	LUL (5), Lingual (2), RUL (4), RLL (2), RML (1)	IBV Valve; Median: 4; Range: 2–6

**Table 3:** Outcomes of endobronchial one-way valve deployment in case series included

First author, year [Article no.]	Outcome of air leak after endobronchial one-way valve placement (%)						Complications related to endobronchial one-way valves	Removal of endobronchial one-way valves n/N, (%)	Days to endobronchial one-way valves removal (days)
	Complete resolution	Days to complete resolution (days)	Reduction	No change	Recurrence	Not reported			
Travaline, 2009 [49]	19 patients (47.5%)	Not specified	18 patients (45.0%)	2 patients (5.0%)	/	1 patient (2.5%)	6 patients: valve expectoration, moderate oxygen desaturation; initial malpositioning of the valve that required redeployment, pneumonia, methicillin-resistant *Staphylococcus aureus* colonization and one unspecified	8/40, (20%)	Mean ± SD: 66 ± 53; Range: 7–143
Gillespie, 2011 [50]	6 procedures (75%)	Mean: 5.2	2 procedures (25%)	/	1 patient (14.3%)	/	/	5/7 (71.4%);	Mean: 37; Range: 14–55
Firlinger, 2013 [51]	10 patients (77%)	1	/	/	3 patients (23%)	/	/	7/13 (53.8%)	Not specified
Reed, 2015 [52]	12 procedures (50%)	Not specified	10 procedures (42%)	/	/	2 procedures (8%)	/	17/21 (81%)	Mean: 57; Range: 1–177
Dooms, 2014 [53]	6 patients (60%)	1	3 patients (30%)	/	3 patients (30%)	1 patient (10%)	/	9/9 (100%)	Median: 23; Range: 14–28
Cordovilla, 2015 [54]	6 procedures (66.7%)	13	/	3 procedures (33.3%)	1 patient (12.5%)	/	/	8/8 (100%)	Median: 49; Range: 8–720
Hance, 2015 [55]	8 patients (57%)	Median: 15; Mean: 29	Not specified	Not specified	4 patients (29%)	/	6 patients: persistent air leak (4) and death^a^ (2)	3/14 (21.4%)	Mean ± SD: 138 ± 84.0; Median: 153
Podgaetz, 2015 [56]	18 patients (94.7%)	9 days in patients with lung metastases; 2 days in the rest patients	1 patient^b^ (5.3%)	/	/	/	/	16/19 (84.2%)	Range: 4–6 weeks
Bakhos, 2016 [57]	6 patients (54.5%)	Mean: 5; Median: 5.8	5 patients (45.4%)	/	1 patient (9.1%)	/	/	8/11 (73%)	Mean ± SD: 70 ± 42; Median: 60 days;
Podgaetz, 2016 [14]	13 patients (100%)	Mean: 5.5; Median:2	/	/	/	/	/	9/13 (69.2%)	4–6 weeks

^a^death not related to deployment of endobronchial one-way valve

^b^valves removed in advance in 1 patient with medical reason

## Discussion

The common treatment for PAL is surgery. However, for those patients with poor cardiopulmonary reserve, the risks of general anesthesia and surgical intervention pose a challenge [35]. Meanwhile, poor wound-healing characteristics could contribute to failure of surgery for PAL such as a low FEV1 percentage, and low maximum voluntary ventilation percentage, steroid use, prior radio-chemotherapy, malnutrition, diabetes, etc. [51, 58]. Previous nonsurgical treatments for PAL have had limited success. Pleurodesis may indirectly impact air leak by creating pleural symphysis and localized inflammatory response, but data is lacking regarding its efficacy [59]. Likewise, autologous blood patch pleurodesis helps to seal air leak with the blood components that can initiate and support a fibrinous and inflammatory response, there is no substantive evidence that it results in earlier resolution of air leak [60].

There is a variety of bronchosopic management of air leak. The first description of bronchoscopic closure of PAL was reported by Hartmann and Rausch in 1977 using tissue glue [61]. Largest case series using glue sealant was reported by Hollaus et al. Forty-five male PAL patients were treated endoscopically for 13 years since 1983 using fibrin glue alone or combination with spongy calf bone depending on size of defects. Air leak was successfully sealed in 16/45 (36%) patients and 2/16 (12% failure rate) recurred [62]. Another category to eliminate air leaks endoscopically is sclerosants. Five patients with visible, central BPF were successfully treated by 100% ethanol injection without complication [63]. 23/35 (66%) patients recovered completely after polidocanol injection without major complication [64]. Different from formers, thermal energy could also be used to treat air leaks. Aynaci et al. reported a successful elimination of air leak by using argon plasma coagulation (APC) which cauterized the area around two BPFs of 1 and 3 mm diameter [65].

A variety of devices employed other than licensed purposes consist of a large category of invasive broncoscopic technique in treating PALs, including Watanabe spigot, vascular occlusion coils, tracheobronchial stents, and Amplatzer occlude devices [66]. Sasada et al. reported a case series of 24 patients treated by Watanabe spigots. Complete resolution of air leak was observed in 12/24 (50%) patients, and a reduction in 7/24 (29%). 5/24 (21%) patients showed no improvement. Complications were as following: migration of the spigot in 4/24(17%) patients; fever, pneumonia, or lung abscess in 5/24 (21%) [67].

**Fig. 2:**
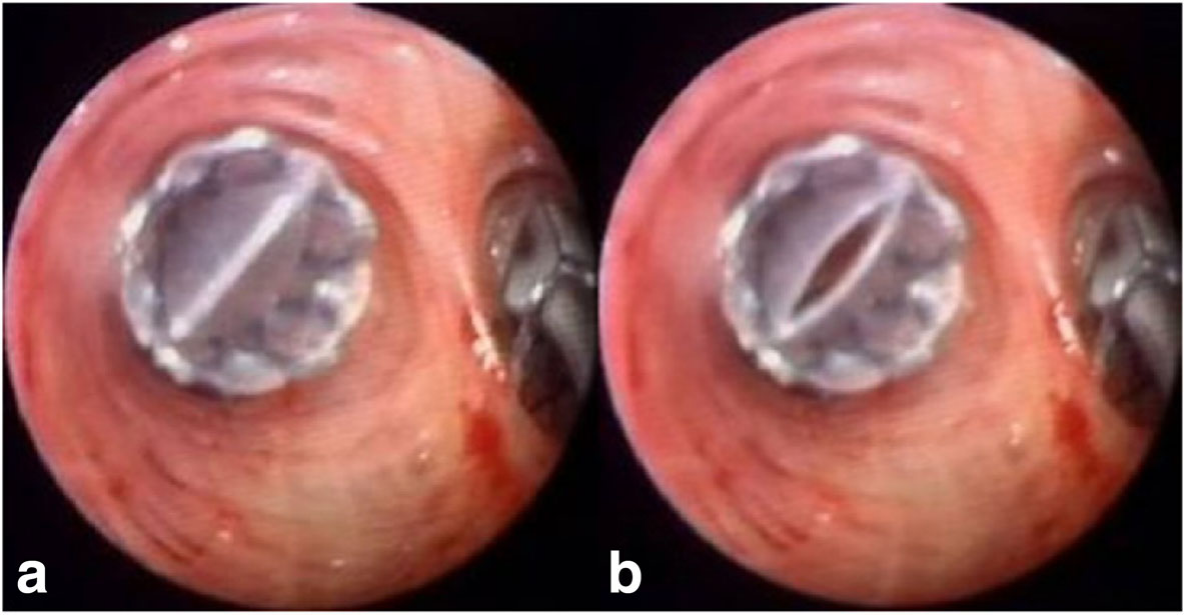
Endoscopic view of one-way valve (**a**) inspiratory phase; (**b**) expiratory phase

Endobronchial one-way valves (Fig. 2) are one of the latest additions to the therapeutic armamentarium for PAL. Since that the interventions using flexible bronchoscopy is possible, it can be performed at the bedside in even the sickest patients [45]. A major benefit of bronchoscopic interventions lies in the fact that they are completely removable. Moreover, endobronchial one-way valves have its advantage for allowing expiration and clearance of distal bronchial secretions, therefore probably reducing the risk of post-obstructive pneumonia [18, 51].

The endobronchial one-way valve system comprises a delivery catheter, a loader system and the implantable valves. Endobronchial one-way valve delivery could depend on either larger lumen of the rigid bronchoscopes or longer and thinner flexible bronchoscopes, the latter becomes the most common choice as it could be easily performed under moderate sedation and could help navigate into more distal bronchi [68]. Former endobronchial one-way valve, the Emphasys® (Emphasys Medical, Inc., Redwood City, CA) is no more commercially circulated. Endobronchial one-way valves are only approved for use in Europe under the name Zephyr® made by Pulmonx while US FDA approved IBV under the name Spiration® made by Olympus. The size of Spiration® IBVs comes to 5 mm, 6 mm, and 7 mm, which is smaller than Zephyr® valves with up to 8.5 mm. It is not equal to compare the number of valves used in the procedure between Zephyr® valves and Spiration® IBV because it could be occurred that instead of using a larger valve to occlude the proximal bronchus, the smaller size would be chosen to be deployed at segmental bronchi to achieve complete occlusion [14].

Although methylene blue has been used for diagnosis of fistulas for many years, it is not frequently used in the treatment of PAL. Methylene blue could be seen within the bronchial tree leading to the fistula following being inserted via the chest tubes. The origin of the air leak could then be identified. Zeller et al. emphasized the use of methylene blue for guiding endobronchial one-way valve deployment. They reported identification of one bronchial segment leading to fistula by methylene blue inserting via chest tube which has not previously been identified by the common balloon method [38]. It could be suggested that it may be also helpful in identification of origin of the intermittent air leak which could not detected by balloon occlusion test.

Another helpful technique in the endobronchial one-way valve deployment procedure is the digital pleural drainage system which is able to continuously monitor and record the air leak in milliliter leakage per minute (ml/min^−1^) providing an objective and reproducible quantitative air leak assessment [32]. Pompili et al. conducted a multicenter international randomized study and showed that, compared with those managed with traditional devices, patients managed with digital drainage systems after lung resections experienced a shorter duration of chest tube placement, shorter hospital stays, and a higher satisfaction rate [69].

Chartis system is originally used to assess collateral ventilation between target lobe and adjacent lobes in endobronchial lung volume reduction. Interestingly, Tian et al. reported a successful treatment of PAL with Zephyr EBVs being placed in bronchus B1 after Chartis system assessment which indicated a significant and constant negative pressure following the catheter being inserted into the bronchus B1 and the balloon sealing the S1 segment [70].

It is believed that continuation of air leaks after valve deployment was due to collateral ventilation between the lobes [56]. Endobronchial lung volume reduction studies have showed underestimated existence of collateral ventilation, or even alveolar-alveolar anastomoses [71]. Absence of atelectasis after lobar exclusion for PAL on chest x-ray is indicative of collateral ventilation which depends on the presence of incomplete fissures [32]. However, Rosell et al. reported a previous reduction of air debit in one patient treated with endobronchial one-way valves proceeding to complete resolution of PAL without further intervention. The simple reduction of the air debit may transform an incontrollable air leak into a situation for conservative management until it heals, and it should not be initially considered a failure [34].

Safety of endobronchial one-way valve implantation could be concluded according to complications listed from the available literature as valve migration or expectoration, moderate oxygen desaturation and infection of related lung. No death related to endobronchial one-way valve implantation has been found in our review. Since that decreased FEV1 after endobronchial one-way valve deployment is inevitable and even immediate respiratory failure has been reported, the patient should be carefully selected before treatment with endobronchial one-way valves in PAL [32, 53].

Endobronchial one-way valve implantation would produce a financial benefit as it can minimize direct hospital costs and eliminate the risks of potential complications. Podgaetz et al. reported that the average cost of the hospitalization before valve implantation was $14,605 including all levels of care. Total cost of procedure, valves, and hospital stay since valve implantation was $13,900 [14]. Dooms and his colleagues reported that in their study the median direct cost related to valve management was €6970 per patient [53]. Santini et al. reported that the mean cost of valve was €4500 whereas the average cost day per day in the intensive care unit in Italy was estimated to be €1500. Thus, a modest reduction in the intensive care unit would justify the use of endobronchial one-way valves as a cost-effective procedure [30].

Although endobronchial one-way valve implantation has now been performed in many countries, most of the studies have limitations of small number of patients included and lack of control. Prospective randomized control studies should be needed to evaluate the effects and safety of endobronchial one-way valve implantation.

## Conclusion

PAL is a common clinical problem with significant morbidity and mortality. It’s possible that traditional treatments are not feasible in high-risk patients and that air leak remains persistent in spite of traditional treatments. The use of endobronchial one-way valve adds to the armamentarium for non-invasive treatments of challenging PAL, especially those with difficulties of anesthesia, poor condition and high morbidity. With its minimal complications, endobronchial one-way valve is a considerable therapeutical option. Nevertheless, prospective randomized controlled trials with large sample should be needed to further evaluate the effects and safety of endobronchial one-way valve implantation in the treatment of PAL.
